# Delivery of Drugs into Cancer Cells Using Antibody–Drug Conjugates Based on Receptor-Mediated Endocytosis and the Enhanced Permeability and Retention Effect

**DOI:** 10.3390/antib11040078

**Published:** 2022-12-19

**Authors:** Toshihiko Tashima

**Affiliations:** Tashima Laboratories of Arts and Sciences, 1239-5 Toriyama-cho, Kohoku-ku, Yokohama 222-0035, Japan; tashima_lab@yahoo.co.jp

**Keywords:** drug delivery system, antibody–drug conjugate, receptor-mediated endocytosis, enhanced permeability and retention effect, solid cancer therapy, cancer antigen, endosomal escape, lysosomal escape

## Abstract

Innumerable people worldwide die of cancer every year, although pharmaceutical therapy has actualized many benefits in human health. For background, anti-cancer drug development is difficult due to the multifactorial pathogenesis and complicated pathology of cancers. Cancer cells excrete hydrophobic low-molecular anti-cancer drugs by overexpressed efflux transporters such as multiple drug resistance 1 (MDR1) at the apical membrane. Mutation-driven drug resistance is also developed in cancer. Moreover, the poor distribution of drug to cancer cells is a serious problem, because patients suffer from off-target side effects. Thus, highly selective and effective drug delivery into solid cancer cells across the membrane should be established. It is known that substances (10–100 nm in diameter) such as monoclonal antibodies (mAbs) (approximately 14.2 nm in diameter) or nanoparticles spontaneously gather in solid tumor stroma or parenchyma through the capillary endothelial fenestration, ranging from 200–2000 nm, in neovasculatures due to the enhanced permeability and retention (EPR) effect. Furthermore, cancer antigens, such as HER2, Nectin-4, or TROP2, highly selectively expressed on the surface of cancer cells act as a receptor for receptor-mediated endocytosis (RME) using mAbs against such antigens. Thus, antibody–drug conjugates (ADCs) are promising anti-cancer pharmaceutical agents that fulfill accurate distribution due to the EPR effect and due to antibody–antigen binding and membrane permeability owing to RME. In this review, I introduce the implementation and possibility of highly selective anti-cancer drug delivery into solid cancer cells based on the EPR effect and RME using anti-cancer antigens ADCs with payloads through suitable linkers.

## 1. Introduction

**Figure 1:**
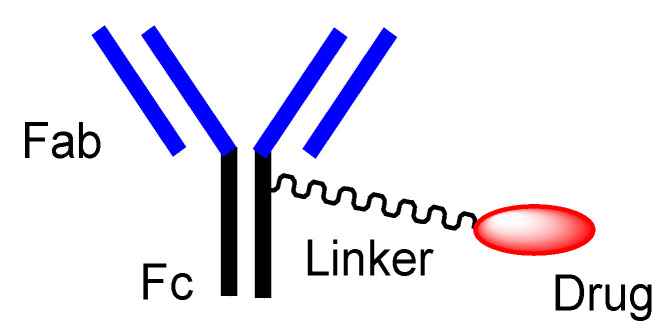
The structure of canonical antibody–drug conjugate (ADC).

Cancer still remains a deadly disease, although pharmaceutical therapy as a key element of medical care has brought better health outcomes in most diseases. Unmet medical needs in oncology should be overcome. Nonetheless, the multifactorial pathogenesis and complicated pathology of cancers confront cancer drug development. At present, cancer immunotherapy using monoclonal antibodies (mAbs) against cancer antigens that are highly selectively expressed on the surface of cancer cells have been developed [1]. However, this strategy based on extracellular attacks by growth factor binding inhibition is an insufficiently efficacious treatment, because it indirectly inhibits growth and does not kill cancer cells promptly. Thus, antibody–drug conjugates (ADCs) (Figure 1) against cancer antigens, particularly with payloads that exhibit their activity in cancer cells based on intracellular attacks through receptor-mediated endocytosis (RME), can be a promising approach to show more effective anti-cancer activity, in addition to the above-mentioned extracellular attacks. Therefore, RME using ADCs against antigens expressed highly selectively on cancer cells can be a solution to kill solid cancers specifically. Moreover, it is known that substances (10–100 nm in diameter), including mAbs, are spontaneously gathered in solid cancers based on the enhanced permeability and retention (EPR) effect [2] due to the underdeveloped lymphatic system in solid tumor parenchyma and high-pressure interstitial fluid in deep cancer tissue. Therefore, ADCs against cancer antigens exhibit anti-cancer effects without off-target side effects based on the EPR effect and RME.

**Figure 2:**
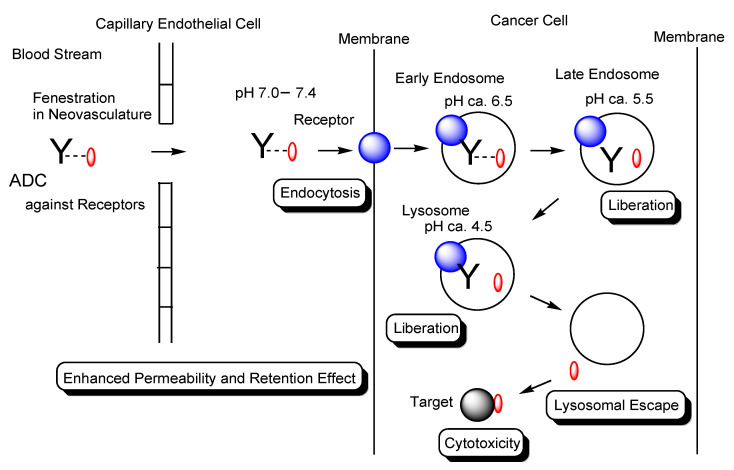
The pathway of intravenously administered antibody–drug conjugates (ADCs) against receptors such as cancer antigens. ADCs were internalized into cancer cells via receptor-mediated endocytosis (RME). Payloads were liberated by pH-sensitive linker cleavage based on acidification as endosome maturation or by enzymatically cleavable linker cleavage based on lysosomal enzymes and were transported to the cytoplasm by endosomal or lysosomal escape via passive diffusion and/or carrier-mediated transporters. Finally, payloads exhibited anti-cancer activity in the cytoplasm or the nucleus. Y represents a monoclonal antibody (mAb). The blue sphere indicates a receptor that mediates endocytosis in cancer cells. The red ovals represent a drug that is tethered with a mAb through a suitable linker. The black sphere indicates target substances such as topoisomerase II or DNA. The dotted line indicates a linker contained in an ADC. The solid line represents the membrane.

In general, the cell membrane permeability of drugs is a serious problem in drug discovery and development. I have introduced several methods for transmembrane drug delivery [3,4,5,6,7,8,9,10], particularly for drug delivery into cancer cells across the membrane via RME using mAbs, cell-penetrating peptides (CPPs), and tumor-homing peptides (THPs) as ligands [5], and for drug delivery to brain cancer cells across the blood–brain barrier (BBB) via receptor-mediated transcytosis (RMT) using ADCs or bispecific mAbs [10]. Solid tumors consist of the parenchyma and stroma. Furthermore, high-pressure interstitial fluid in deep cancer tissue and an underdeveloped lymphatic system in solid tumor parenchyma prevent substance movement. Well-defined drug design should be conducted in compliance with such physically and biologically systematic structures based on structuralism. Compounds are divided into three categories in size: low-molecular compounds (molecular weight (MW) < approximately 500 Da), high-molecular compounds (MW > approximately 3000 Da), and middle-molecular compounds (MW from approximately 500 Da to approximately 3000 Da). Large molecules cannot go through narrow pores. Water and oil do not mix. According to size and hydrophobicity, the behavior of compounds is subject to systematic structures ruled by structuralism. mAbs are high-molecular compounds and thus cannot cross the bilayer lipid membrane via passive diffusion. They enter cells via RME or macropinocytosis [5,10]. On the other hand, hydrophobic low-molecular compounds cross the bilayer lipid membrane via passive diffusion but are substrates of efflux transporters such as multiple drug resistance 1 (MDR1) (P-glycoprotein). Hydrophilic low-molecular compounds enter cells via carrier-mediated transport using solute carrier (SLC) transporters that mediate substrate-specific transportation [3]. Therefore, the well-designed compounds would be pharmacokinetically controlled based on structural pharmaceutical science established by scientific materialism according to structuralism. In this perspective review, I introduce updated highly selective anti-cancer drug delivery into solid cancer cells across the membrane, focusing on the usage of ADCs via the EPR effect and subsequent RME (Figure 2).

## 2. Discussion

### 2.1. Effective and Non-Invasive Cancer Cures

Cancer, also called a neoplasm or malignant tumor, is a serious disease with abnormal cell proliferation and metastasis to other nearby tissues. Nearly 10 million people globally died of cancer, accounting for one in six deaths, in 2020 [11]. Cancer pathogenesis and progression are so complicated that their pathological mechanisms remain unclear. Therefore, an innovative treatment methodology should be developed to satisfy unmet medical needs. The current methods for the treatment of solid cancers are surgical remedy, radiotherapy, chemotherapy, and immunotherapy. However, chemotherapy using anti-cancer agents has critical problems such as cancer drug resistance and off-target side effects [5]. As a cause of cancer drug resistance, it is well-known that efflux transporters such as MDR1 are highly overexpressed at the apical membrane of cancer cells. Low-molecular anti-cancer drugs are excreted from the cancer cell membrane to the outside of cancer cells [5]. As a result, they readily efflux from cancer cells and are rendered ineffective [5]. Thus, overwhelming drug delivery methods into cancer cells across the membrane are needed. Moreover, genetic alteration due to rapid and highly frequent cell proliferation induces a variety of phenotypes which could overcome the original pharmaceutical activities at the molecular level [12]. Nausea, hair loss, and bone marrow suppression are representative off-target side effects. Generally, anti-cancer drugs exhibit their cytotoxicity not only in cancer cells but also in normal cells, because the biological mechanisms that anti-cancer drugs inhibit, such as mitosis inhibition, are present even in normal cells. Thus, highly selective drug distribution methods for cancer cells are also needed. Well-defined drug design must be established for patients’ quality of life. Eventually, pharmacokinetically scrupulous approaches to implement highly selective anti-cancer drug delivery into solid cancer cells across the membrane should be developed, which is regulated through physically and biologically systematic structures based on the structuralism proselytized by Dr. Claude Lévi-Strauss [13,14].

It is known that cancer cell-specific molecules known as cancer antigens such as EGFR (colorectal cancer), GD2 (neuroblastoma), HER2 (breast cancer), VEGFR2 (gastric cancer), Nectin-4 (bladder cancer), or TROP2 (triple-negative breast cancer) are expressed on the surface of cancer cells (Table 1) [1]. They are essentially involved in cancer cell activity. Intriguingly, some of them induced RME after binding to their ligands. Such RME can be a solution for both the problems of cancer cell selectivity and membrane permeation at the same time.

### 2.2. Endocytosis and Endosomal–Lysosomal System

Cells absorb high- and middle-molecular compounds via endocytosis, which induces the plasma membrane invagination to form endosomes containing substances essential for homeostasis. On the other hand, they absorb low-molecular compounds via carrier-mediated transport using SLC transporters and/or via passive diffusion and minerals via carrier-mediated transport using ion channels and/or using ion pumps. However, detailed endocytosis mechanisms are poorly understood. Based on the trigger types, endocytosis is broadly divided into (i) RME, (ii) nonreceptor-mediated endocytosis, and (iii) caveolae-mediated endocytosis. Moreover, based on the invagination type, endocytosis is broadly divided into (a) clathrin-dependent endocytosis (endosomal diameter of 85–150 nm), (b) caveolae-dependent endocytosis (endosomal diameter of 50–100 nm), (c) clathrin- and caveolae-independent endocytosis (endosomal diameter of approximately 90 nm), (d) macropinocytosis (endosomal diameter of 0.2–5 μm), and (e) other endocytoses that occur in a fairly unexpected manner [5]. Since several mechanisms of endocytosis can happen at the same time, the substance trajectories via endocytosis are complicated. However, clathrin-dependent endocytosis is a predominant mechanism among a variety of endocytosis processes. Anti-HER2 bispecific mAbs targeting two non-overlapping epitopes on HER2 in domain IV and domain II [15], Sym004 as the mixture of two anti-EGFR mAbs [16], and Brentuximab vedotin as a CD30-targeted ADC [20] were internalized into cells via clathrin-dependent endocytosis. When ligands bound to their corresponding receptors on the cell surface, the clustering of ligand–receptor–AP2 (adaptor protein complex-2) complexes induced intracytoplasmic AP2–clathrin interaction and eventually formed clathrin-coated vesicles constricted by dynamin and actin. After scission, such clathrin-coated vesicles became endosomes through uncoating and then were subject to the endosomal–lysosomal system [45].

Endosomes formed through the plasma membrane invagination are sorted by Rab proteins, dependent on their contents, to the secretory pathway leading to exocytosis or to the degradation pathway leading to lysosomal degradation [6]. The pH in endosomes gradually decreases, as endosomal maturation, from approximately 6.5 in the early endosome, to approximately 5.5 in the late endosome, and, finally, to approximately 4.5 in lysosomes by vacuolar H^+^-ATPase proton pumps. The endosomal–lysosomal system plays a vital role in pharmacokinetics with respect to transportation, pH- or enzyme-sensitive payload release from ADCs, pH-sensitive ligand–receptor disassociation, and the FcRn-mediated transient salvation of Abs from lysosomal degradation. Thus, well-established drug design for cancer therapy should take advantage of such endosomal–lysosomal machinery as physically and biologically systematic structures.

### 2.3. Ab Drugs for Cancer Therapy

At present, bio-pharmaceuticals including mAb drugs dominate the pharmaceutical market. Abs are immunoglobulin (Ig) proteins that recognize and neutralize specifically foreign substances in the immune system and are categorized into IgG, IgA, IgM, IgD, and IgE [46]. An IgG Ab, the most abundant in the blood among Ig proteins, is structurally constructed from two heavy chains and two light chains and is enzymatically divided into a fragment antigen-binding (Fab) region and a fragment crystallizable (Fc) region (Figure 1). Fc region as a ligand binds several types of Fc receptors (FcRs) such as the neonatal Fc receptor (FcRn) and Fc-gamma receptors (FcγRs). Salvation from lysosomal degradation based on FcRn in endothelial cells elongates the biological half-life of Abs and enlarges their volume of distribution [8]. Although Abs do not bind the FcRn under physiological pH, endocytosed bystander Abs bind the FcRn in endosomes under weak acidic conditions [8]. Ab–FcRn complexes in endosomes are exposed to the systemic circulation via the fusion between endosomes and the apical membrane in the secretory pathway, without being degraded in lysosomes in the degradation pathway. Exposed Ab–FcRn complexes dissociate under physiological pH [8]. Eventually, Abs whose Fc region is not occupied with components to interfere with FcRn binding, such as linkers or payloads, have a long biological half-life. The half-lives were 29.7 days for IgG1, 26.9 days for IgG2, and 15.7 days for IgG3 [47]. Ab glycosylation plays an important role in maintaining the structure and function of the Ab. The removal of glycosylation abolishes FcR binding and antibody-dependent cellular cytotoxicity (ADCC). In particular, sialylation in the Fc domain elongates the half-life in serum [48,49,50,51].

The RGD (Arg–Gly–Asp) sequence of ligands as THPs that are oligopeptides with the inherent property of recognizing the tumor cells specifically binds ανβ3 and ανβ5 integrins and induces biological events, including endocytosis [5]. The NGR (Asn–Gly–Arg) sequence of ligands binds to the receptor aminopeptidase N [5]. Furthermore, cancer cell surfaces are negatively charged. CPPs such as TAT (YGRKKRRQRRR) and penetratin (RQIKIWFQNRRMKWKK) are positively charged short peptides with 5–30 amino acids, which are internalized into cells across the membrane through RME or direct translocation. Thus, positively charged CPPs electrostatically interact with negatively charged cancer cell surfaces [5]. Subsequently, electrostatically gathered CPPs, as a ligand, bind negatively charged heparan sulfate proteoglycans, as a receptor, and, subsequently, are internalized into cancer cells through RME [5]. Nonetheless, THPs and CPPs lack cell selectivity, compared to Abs. Abs should be adopted as a ligand due to their high selectivity, to avoid off-target side effects. The concept of the “magic bullet” proselytized by Dr. Paul Ehrlich in 1900 is possible by mAbs against cancer antigens [52].

It is known that highly selective molecules as cancer antigens are expressed on the surface of cancer cells. Ab drugs targeting extracellular cancer antigens can be used for cancer therapy, according to anti-cancer effects such as ADCC, complement-dependent cytotoxicity (CDC), antibody-dependent cellular phagocytosis (ADCP), and cell growth inhibitory activity based on growth factor binding inhibition. In fact, most clinical trials using Ab drugs, that is, more than 2200 trials, were performed for cancers before 2014. As a result, the number of clinically approved Ab drugs for the treatment of cancers was the largest in 2014, followed by autoimmune diseases/autoinflammatory diseases as the second largest disease group [53]. However, there were not so many approved Ab drugs for cancers, of which there were only 15–20, although a lot of clinical trials were conducted. This suggests that just blocking cancer antigens by Abs did not reach an effective level of cytotoxic action in cancer cells in many cases. Nonetheless, promising Ab drugs were developed. Anti-PD-1 (programmed cell death 1) Abs and anti-PD-L1 (programmed death ligand 1) Abs, as immune checkpoint inhibitors, block the binding between PD-L1 on cancer cells and PD-1 on T cells [54]. The binding of PD-L1, as a checkpoint protein, to PD-1, as a checkpoint protein, suppressed T cells. Thus, inhibiting PD-1 or PD-L1 allowed T cells to kill cancer cells. Nivolumab (Opdivo^®^) [17], as an anti-PD-1 mAb, was approved for metastatic lung squamous cell carcinoma in 2014 by the FDA. The other FDA-approved mAbs for cancers were launched to market [55,56]. Particularly, in the 2020s, four mAbs were approved by the FDA (Table 1) [18]: (i) isatuximab (Sarclisa^®^) against CD38 for multiple myeloma in 2020, (ii) tafasitamab (Monjuvi^®^) against CD19 for diffuse large B cell lymphoma in 2020, (iii) naxitamab (Danyelza^®^) against GD2 for high-risk neuroblastoma and refractory osteomedullary disease in 2020, and (iv) dostarlimab (Jemperli^®^) against PD-1 for endometrial cancer in 2021. It is evident from their approval status that drug development is being shifted from mAb drugs to ADCs. A more effective cancer therapy not only by Abs but also by payloads is expected for ADCs.

### 2.4. ADCs for Cancer Therapy

**Figure 3:**
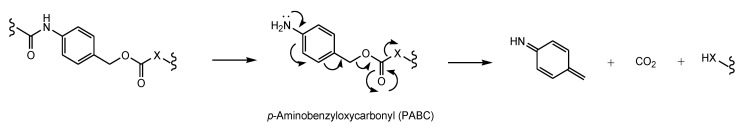
The self-immolative mechanism of *p*-aminobenzyloxycarbonyl (PABC). X represents an arbitrary heteroatom such as N or O.

**Figure 4:**
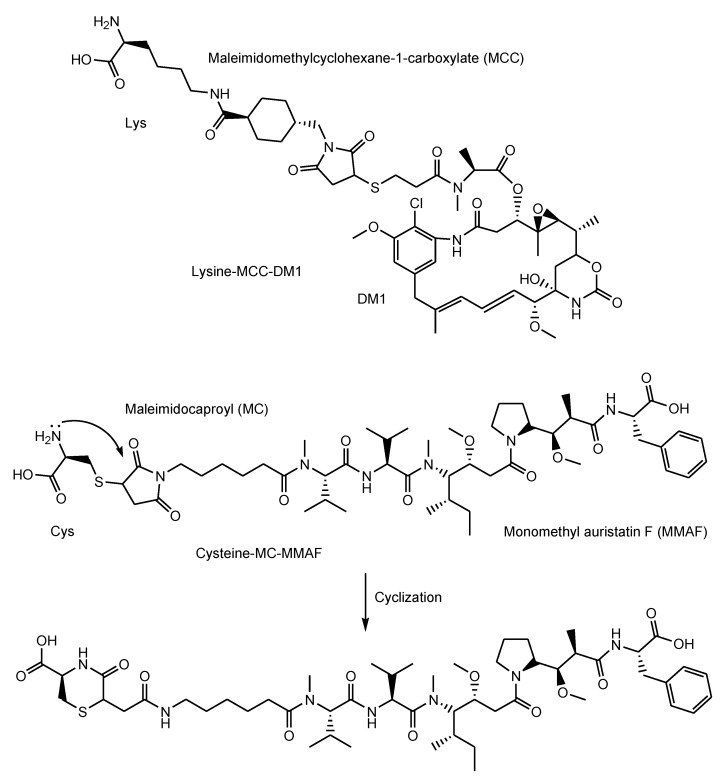
The structures of lysine-MCC-DM1 and cysteine-MC-MMAF as catabolites in lysosomes, and the cyclic form of cysteine-MC-MMAF.

Abs exhibit specific binding to epitopes as antigens. Thus, Abs can demonstrate a highly selective distribution to the cancer cells based on cancer antigens. Drug delivery into the brain across the membrane can be carried out via RMT using anti-receptor mAbs with cargos [57]. Similarly, mAbs, as vectors, particularly ADCs, can deliver payloads into cancer cells across the membrane. Structurally, canonical ADCs consist of mAbs and payloads through suitable linkers that are non-cleavable or cleavable (Figure 1). Several classes of cleavable linkers for ADCs have been developed [58]: (i) pH-sensitive cleavable linkers such as hydrazones, (ii) reductively cleavable linkers, (iii) enzymatically cleavable linkers such as cathepsin B-labile Val-citrulline (Cit) dipeptide, (iv) self-immolative linkers such as *p*-aminobenzyloxycarbonyl (PABC) (Figure 3) [59], and (v) other mechanistically cleavable linkers. Moreover, intriguingly, although the maleimidomethylcyclohexane-1-carboxyl (MCC) linker is a non-cleavable linker, lysine-MCC-DM1 (MW1089.69) (Figure 4) was isolated by lysosomal enzymes and transferred from lysosomes to the cytoplasm via carrier-mediated transport using the lysosomal transporter SLC46A3 [23]. The steric hindrance of mAbs inhibits the activity expression of linked payloads. Thus, ADCs do not show cytotoxic activity under normal conditions.

**Figure 5:**
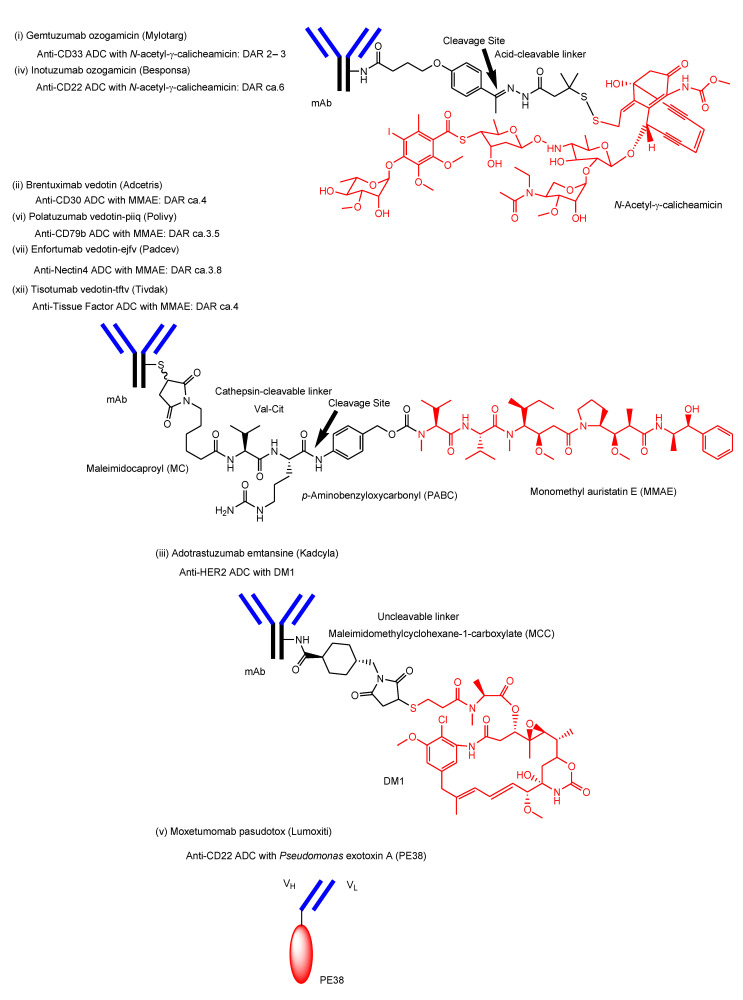

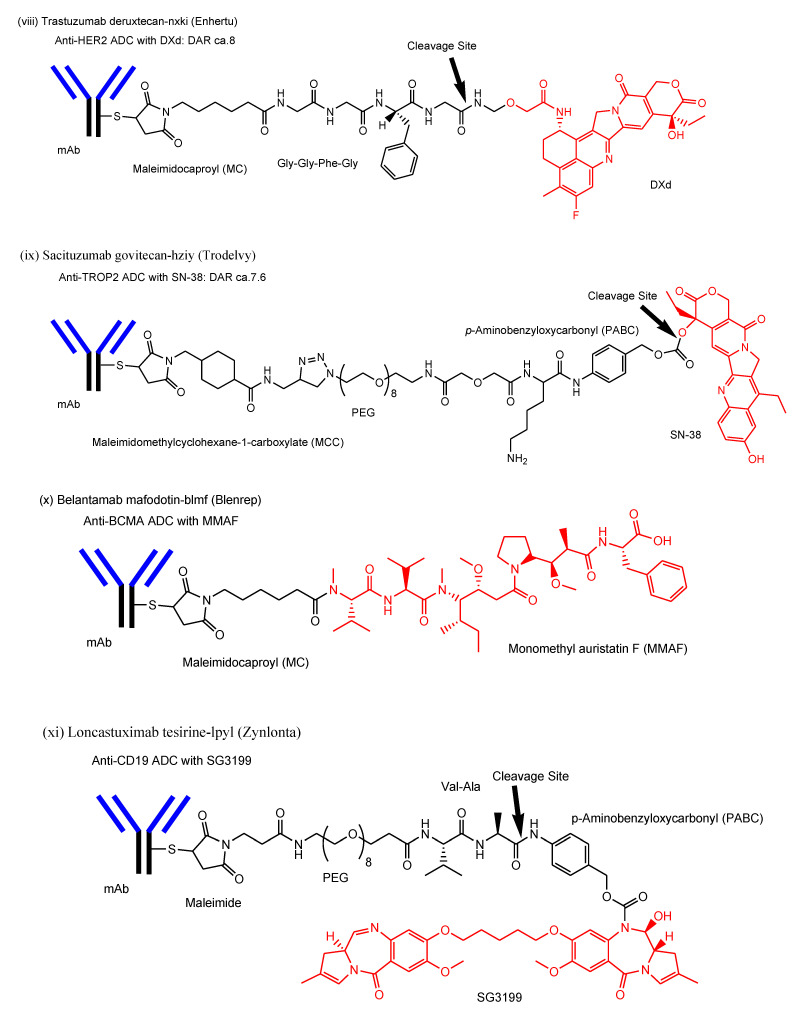
The structures of the FDA-approved antibody–drug conjugates (ADCs) through suitable linkers. DAR stands for drug-to-antibody ratio. (i) Gemtuzumab ozogamicin (Mylotarg^®^) and (iv) polatuzumab vedotin-piiq (Polivy^®^) have the same payload (*N*-acetyl-γ-calicheamicin) and linker (acid-cleavable linker). (ii) Brentuximab vedotin (Adcetris^®^), (vi) polatuzumab vedotin-piiq (Polivy^®^), (vii) enfortumab vedotin-ejfv (Padcev^®^), and (xii) tisotumab vedotin-tftv (Tivdak^®^) have the same payload (monomethyl auristatin E (MMAE)) and linker (enzymatically cleavable linker). ADCs with the same payload–linker systems share the same mechanisms of payload endosomal/lysosomal escape.

So far, 12 ADCs have been clinically approved by the FDA (Figure 5) (Table 1) [60]. All of them are developed for cancer therapy. Their payloads, such as microtubule inhibitors, mechanically show their anti-cancer activity in cancer cells. Thus, they must enter cancer cells across the membrane.

For the 12 ADCs arranged below in order of approval and structurally shown in Figure 5, where the Roman numerals ((i)–(xii)) correspond to each other in the text and Figure 5, it turns out that endocytosis and endosomal/lysosomal escape are needed by them to show their anti-cancer activities. Payloads and linkers are shared in ADCs, resulting in them sharing the same endosomal/liposomal escape modes.

i. Gemtuzumab ozogamicin (Mylotarg^®^) against CD33, approved for blood cancer in 2000 and 2017, was endocytosed through the endosome/lysosome pathway in CD33-expressing HL-60 cells [19]. The endosomal and/or lysosomal escape mechanism of *N*-acetyl-γ-calicheamicin (MW approximately 1410) was unclarified. Calicheamicin γ1, structurally related to *N*-acetyl-γ-calicheamicin, showed a 45-fold less efficient cleavage of cellular DNA at 0 °C, compared to 37 °C, due to poor cell permeability at a low temperature [61]. Thus, it was suggested that *N*-acetyl-γ-calicheamicin was unlikely to cross the plasma membrane via passive diffusion, while it might cross the membrane energy-dependently via carrier-mediated transport or via a type of endocytosis. Since the sugar residues of *N*-acetyl-γ-calicheamicin are involved in DNA interaction, it would retain sugar residues and alkynes after endosomal and/or lysosomal escape. Probably, *N*-acetyl-γ-calicheamicin is structurally stable under weak acid because of the usage of an acid-cleavable linker. Carrier-mediated transport might be its escape mechanism, although its molecular weight is relatively large, compared to lysine-MCC-DM1 (MW1089.69).
ii. Brentuximab vedotin (Adcetris^®^) against CD30, approved for blood cancer in 2011, was internalized via endocytosis [21]. Enzymatically released monomethyl auristatin E (MMAE) was transported to the cytoplasm after lysosomal escape via passive diffusion [22].
iii. Adotrastuzumab emtansine (Kadcyla^®^) against HER2, approved for solid cancers such as breast cancer in February 2013, entered the cell via RME and released lysine-MCC-DM1 (Figure 4) as a catabolite to the cytoplasm through carrier-mediated transport using the lysosomal transporter SLC46A3 [54] in the lysosomal escape process [24].
iv. Inotuzumab ozogamicin (Besponsa^®^) against CD22 with an acid-cleavable linker, approved for blood cancer in 2017, was internalized into cells and released a potent cytotoxic agent, *N*-acetyl-γ-calicheamicin, to the cytoplasm and the nucleus after lysosomal escape [25].
v. Moxetumomab pasudotox-tdfk (Lumoxiti^®^) against CD22, approved for blood cancer in 2018, was internalized through clathrin-coated pits into the endocytic compartment. This is structurally an anti-CD22 immunoglobulin variable domain genetically joined to *Pseudomonas* exotoxin (PE38) as a payload, although it is not a canonical ADC. PE38 was cleaved by the disulfide bond reduction in the endosome and was released to the cytoplasm by way of the endoplasmic reticulum [26]. It was unknown what the endosomal escape of the cleaved PE38 was like. In the future, different formats of Ab fragments and their derivatives, such as nanobodies (approximately 15 kDa) known as single-domain Abs or variable fragments of heavy-chain (VHH) domains, will be developed in ADCs [62].
vi. Polatuzumab vedotin-piiq (Polivy^®^) against CD79b with an enzymatically cleavable linker, approved for blood cancer in 2019, entered cells and released a potent cytotoxic agent, MMAE, into the cytoplasm after lysosomal escape via passive diffusion [27].
vii. Enfortumab vedotin-ejfv (Padcev^®^) against Nectin4, approved for solid cancers such as urothelial cancer in 2019, was intracellularly internalized by endocytosis and was degraded in a lysosome to subsequently release the cytotoxic payload MMAE [28].
viii. Trastuzumab deruxtecan-nxki (Enhertu^®^) against HER2, approved for solid cancers such as breast cancer in 2019, underwent endocytosis by binding to HER2-positive tumor cells and released the payload deruxtecan (DXd) by lysosomal cathepsins [29]. DXd demonstrated passive diffusion across the membrane [63].
ix. Sacituzumab govitecan-hziy (Trodelvy^®^) against TROP2, approved for solid cancers such as breast cancer in 2020, was internalized via RME. The payload SN-38 was released by double ester hydrolysis of the CL2A linker at low pH within lysosomes [30,31,32]. It was revealed that SN-38 crossed the plasma apical membrane via carrier-mediated transport using transporters different from organic anion-transporting polypeptides (OATP) and the monocarboxylate transporter (MCT) in Caco-2 cells [64]. Thus, it was suggested that SN-38 was transported from lysosomes into the cytoplasm via carrier-mediated transport.
x. Belantamab mafodotin-blmf (Blenrep^®^) against BCMA, approved for blood cancer in 2020, was probably endocytosed clathrin-dependently by binding cell-surface BCMAs. It was supported by the fact that it possessed the cytotoxic payload auristatin F (MMAF) [33]. MMAF was released via proteolytic cleavage, as cysteine-maleimidocaproyl (MC)-MMAF (Figure 4) that became further the six-membered cyclic form derived from cysteine and maleimido by the intramolecular nucleophilic substitution of the amino group to the ketone (Figure 4) [65]. Positively charged cysteine-MC-MMAF under physiological pH is not thought to be membrane-permeable via passive diffusion [66]. Similarly, it would be positively charged under a pH of approximately 4.5 in lysosomes. However, the cyclic form of cysteine-MC-MMAF lost the amino group and could be transported across the lysosomal membrane via passive diffusion, although cysteine-MC-MMAF might be a substrate of arbitrary lysosomal transporters such as SLC46A3 for lysine-MCC-DM1.
xi. Loncastuximab tesirine-lpyl (Zynlonta^®^) against CD19, approved for blood cancer in 2021, was internalized via RME and released the cytotoxic molecule SG3199 by lysosomal proteolysis and the subsequent self-motivated degradation of the linker [34]. MDR1 decreased the permeability of pyrrolobenzodiazepin (PBD) dimers such as SJG-136 and DRG-16, which were structurally related derivatives of SG3199, across the cell membrane in Caco-2 cells [67]. Thus, SG3199 was suggested to be transported across the membrane via passive diffusion.
xii. Tisotumab vedotin-tftv (Tivdak^®^) against Tissue Factor, approved for solid cancers such as cervical cancer on 20 September 2021, was thought to be endocytosed and released the cytotoxic payload MMAE [35].

All approved ADCs enter cancer cells via RME, which reflects the fact that payloads with the activity in the cytosol or the nucleus should be delivered into cancer cells across the membrane. On the other hand, ADCs possessing payloads with activity outside of cancer cells are not necessary to be internalized via RME. Instructively, the basic prevailing strategy for ADCs can be inferred from approved ADCs that are all internalized into cells via RME. However, no ADCs had been approved by the FDA in fourth quarter of 2021 and in the first half of 2022 [68,69]. Accordingly, novel ADCs have not been recently launched on the market. At present, many typical ADCs are under clinical trials. As representative cases, (α) Dato-DXd as an anti-ROP2 ADC [36], (β) HER3-DXd as an anti-HER3 ADC [37], (γ) DS-7300 as an anti-B7-H3 ADC [38], (δ) DS-6000 as an anti-CDH6 ADC [34], and (ε) DS-3939 as an anti-TA-MUC1 ADC [39] were evaluated in clinical trials using DXd as a payload based on the strategy of trastuzumab deruxtecan-nxk. (ζ) BYON3521 as an anti-c-MET receptor ADC with duocarmycin via a cathepsin-cleavable linker (ClinicalTrials.gov Identifier (accessed on 19 October 2022): NCT05323045), (η) STI-3258 as an anti-Trop2 ADC (NCT05060276), (θ) STRO-002 as an anti-folate receptor α ADC with 3-aminophenyl hemiasterlin via a cathepsin-cleavable linker (NCT03748186), (ι) ABBV-085 as an anti-leucine-rich repeat containing 15 (LRRC15) ADC with MMAE via a cathepsin-cleavable linker (NCT02565758), and (κ) STI-6129 as an anti-CD38 ADC with duostatin 5.2 via a non-polyethylene glycol linker (NCT05584709) are in phase 1 for solid tumors. Moreover, (λ) ARX788 as an anti-HER2 ADC with MMAF via a non-natural amino acid linker (NCT04983121), (μ) MORAb-202 as an anti-folate receptor α ADC with eribulin via a cathepsin-cleavable linker (NCT05577715), (ν) SYD985 as an anti-HER2 ADC with duocarmycin via a cathepsin-cleavable linker (NCT04205630), (ξ) RC48 (disitamab vedotin) as an anti-HER2 ADC with auristatin E via a cathepsin-cleavable linker (NCT04329429), and (ο) MRG002 as an anti-HRE2 ADC with MMAE via a cathepsin-cleavable linker (NCT05263869) are in phase 2 for solid tumors. (π) XMT-1536 (upifitamab rilsodotin) as an anti-NaPi2b ADC with auristatin F via a hydrophilic polymer linker (NCT05329545) and (ρ) IMGN-853 (mirvetuximab soravtansine) as an anti-folate receptor α ADC with DM4 via a disulfide-containing cleavable linker (NCT04296890) are in phase 3 for solid tumors. Furthermore, a lot of clinical trials including formats of Ab fragments and their derivatives, such as a single-chain variable fragment (scFv), have been performed [70,71]. Nevertheless, ADCs for blood cancers are predominant. It is true that it is difficult for anti-cancer drugs to enter deep inside solid cancer tissues, but ADCs for solid cancers continue to be developed from clinical trials. In ADC development, breakthrough solutions on the matter are demanded to be created under present static conditions. The EPR effect is one of such solutions to develop ADCs for solid cancer therapy.

### 2.5. EPR Effect

**Figure 6:**
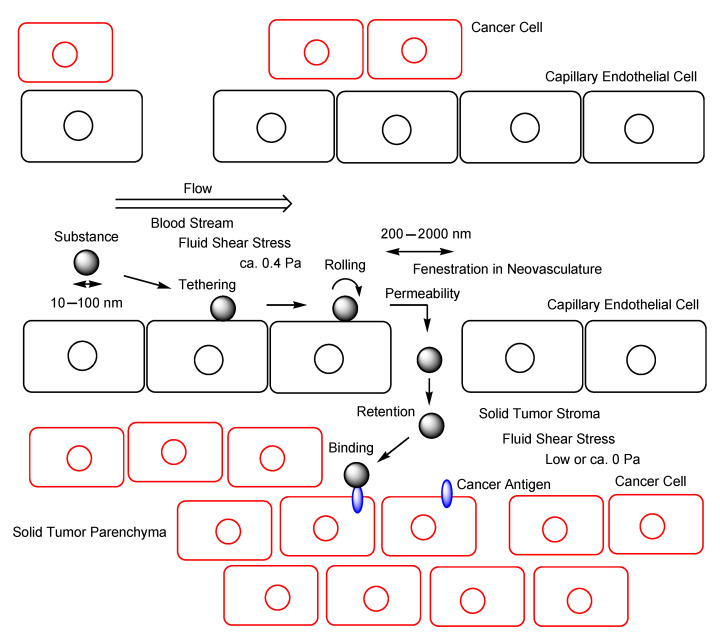
The mechanism of the enhanced permeability and retention (EPR) effect. Substances (10–100 nm in diameter) such as monoclonal antibodies (mAbs) or nanoparticles spontaneously penetrate into solid tumor stroma or parenchyma through the capillary endothelial fenestration, ranging from 200 to 2000 nm, in neovasculature, caused by vascular endothelial growth factor (VEGF) secreted from cancer cells, due to wall fluid shear stress (FSS) (approximately 0.4 Pa), and, subsequently, remain there because of an underdeveloped lymphatic system in solid tumor parenchyma and high-pressure interstitial fluid in deep cancer tissue.

It is well-known that nanoparticles (10–100 nm in diameter) in systemic circulation are spontaneously gathered in solid cancers [2], although there are various opinions on the suitable nanoparticle size. This phenomenon is called the EPR effect (Figure 6) [72], found by Dr. Yasuhiro Matsumura and Dr. Hiroshi Maeda in 1986 [73], and is of current interest as passive targeting for cancer therapy using nanoparticles such as Doxil^®^. An IgG molecule often used for ADCs is approximately 14.2 nm in diameter (approximately 150 kDa). Numerically, ADCs are barely potent enough to receive the benefits of the EPR effect. Blood vessel permeability is enhanced in tumor vessels that branch de novo from the existing vascular system using VEGF secreted from cancer cells, due to a defective and leaky vasculature that easily supply enough nutrition and oxygen to cancer cells through gaps between the endothelial cells, compared to normal vessels. Actually, neovasculatures induced by VEGF are fenestrated [74]. The endothelial fenestrations of new blood vessels are of dimensions ranging from 200 to 2000 nm [75]. Physically, nanoparticles pass into solid tumor parenchyma through a type of sieve of a defective and leaky vasculature. An irregular blood stream in tumor vessels, an underdeveloped lymphatic system in solid tumor parenchyma, and high-pressure interstitial fluid in deep cancer tissue also enable nanoparticles to remain in solid tumor parenchyma.

The transport of ADCs through gaps in the leaky vasculature into solid tumor parenchyma reminds me of the double-slit experiments for photons or electrons, which suggested the wave–particle duality of matter due to the formation of an interference pattern. When the intravenous single-molecule administrations of arbitrary ADCs are conducted, the trajectories to the target cancer tissue based on the systemic circulation and the EPR effect are different in each trial, dependent on the presence or absence of salvation by the FcRn, the interaction with plasma proteins and immune cells, and other factors such as which blood vessels are passed through, which cells are interacted with, and which leaky gap they fall into based on the EPR effect. Their actual routes on the way to the cancer tissue cannot be determined. Their behaviors might be based on the molecular disorder according to the principle of statistical mechanics proselytized by Dr. Ludwig Boltzmann. Furthermore, labeled ADCs for localization imaging could follow a route different from that of non-labeled original ADCs. Their behaviors might act according to the uncertainty principle proselytized by Dr. Werner Heisenberg in quantum mechanics. These phenomena resemble a quantum. However, ADCs do not possess wave properties, but particle properties, due to their own large molecular size and non-negligible interaction with an infinite number of other molecules such as water and proteins that enable apparently counterfeit diffraction coming around behind fenestrations in a non-vacuum. Therefore, the pharmacokinetics of ADCs are regulated by fluid mechanics rather than quantum mechanics.

Nanoparticles are subject to tethering and rolling on the surface of endothelial cells, without forming sediments and aggregates and the subsequent attachment to ligand molecules expressed on their surface by fluid shear stress (FSS) under dynamic conditions in the systemic circulation. The cellular uptake of SiO_2_ nanoparticles (50 nm in diameter) via endocytosis was more greatly enhanced under low FSS (0.05 Pa, 1 dyn/cm^2^ = 0.1 Pa), compared to under static conditions (0 Pa) and under high FSS (0.5 Pa), respectively, in in vitro assays using human umbilical vein endothelial cells (HUVECs) [76,77]. Furthermore, Angiopep-2 is a ligand of low-density lipoprotein receptor-related protein (LRP). Angiopep-2-loaded liposomes (80–95 nm in diameter) were transported 2.7-fold under low FSS (0.1 Pa) and 3.5-fold under high FSS (0.6 Pa) than with static incubation (0 Pa), using the model microfluidic BBB. However, Angiopep-2-loaded liposomes were tethered and subsequently were more greatly internalized under low FSS (0.1 Pa) or after static incubation (0 Pa) than under high FSS (0.6 Pa) using the brain endothelial cells [78]. Interestingly, these findings imply that high FSS (0.6 Pa) evokes transport via the paracellular route through the leakage between cellular junctions. The average wall FSS is approximately 0.4 Pa in normal microvessels [79]. Thus, it was suggested that FSS in normal microvessels substantially drove nanoparticles to be gathered through leaky vasculature in solid cancers based on the EPR effect. The EPR effect relied not only statically on leaky vasculature and stagnant conditions in solid tumor parenchyma, but also dynamically on FSS by the blood stream (Figure 6).

Nonetheless, the collision frequency and attachment level between ADCs, conveyed through defective and leaky vasculature, and ligand molecules, expressed on the surface of solid cancer cells, might be decreased under static, stagnant conditions in solid tumor parenchyma with an underdeveloped lymphatic system. Moreover, ADCs might bind collagen IV or fibrin in the case of stroma-rich tumors due to the stromal barrier [80]. On the other hand, such a collision frequency and attachment level might be increased in blood cancer due to a weakened FSS in the blood stream of the systemic circulation, because bone marrow is a semi-solid tissue full of blood and abnormal blood cancer cells also circulate in the blood stream together with ADCs. These considerations are not likely to contradict the developmental status of ADCs for blood cancer and solid cancers. In order to increase the collision frequency and attachment level in solid tumor parenchyma, the number of ADC molecules can be increased, the ADC molecular size can be increased, or solid tumor parenchyma can be made leakier.

### 2.6. Implementation of EPR Effect

Doxil^®^ is a doxorubicin-encapsulated liposome coated with polyethyleneglycol (PEG) (approximately 80 nm in diameter [40]). Doxorubicin is a topoisomerase II inhibitor. PEGylation on liposomes avoids capture by the reticuloendothelial system in the liver and spleen and, consequently, enables the long-term retention in the systemic circulation. Doxil^®^ gathers in interstitial cavities of solid cancer tissues, such as ovarian cancer and breast cancer, based on the EPR effect and releases doxorubicin molecules there. At present, Doxil^®^ is clinically used for the treatment of ovarian cancer and breast cancer [41]. Although doxorubicin transports the membrane through passive diffusion [81], it is a substrate of MDR1 [82]. MDR1s are overexpressed on cancer cells, compared to normal cells. Thus, payloads such as doxorubicin should be preferably released inside cancer cells to effectively destroy cancer cells. Interestingly, the anti-EGFR and anti-PEG bispecific Ab, that is, PEG engager^EGFR^, bound both PEGylated liposomes containing doxorubicin (Doxisome) and EGFRs and showed antiproliferation activity in in vitro assay using EGFR-positive cancer cells [42]. It was thought that crosslinking EGFRs by PEG engager^EGFR^ molecules on PEGylated liposomes induced endocytosis [42]. Crosslinking was suggested to invoke endocytosis [83,84]. Synthetic nanoparticles, as carriers, are also often used for the treatment of cancer based on the EPR effect. Specific antibodies on nanoparticles are being developed [85].

### 2.7. Promising ADCs for Cancer Therapy

To make a success of ADCs for cancer therapy, there are three points to overcome. First, ADCs gathered in solid tumor parenchyma based on the EPR effect should tether cancer antigens as receptors on the surface of cancer cells, even under static, stagnant conditions. Second, ADCs with payloads that exhibit their activity in the cytoplasm or the nucleus should be internalized into cancer cells across the plasma membrane via RME. Third, released payloads should escape from endosomes or lysosomes to the cytoplasm.

This first problem is a relatively challenging task. It is thought that the FSS at static, stagnant regions in solid tumor parenchyma is probably almost close to 0 Pa, or very little, whereas the cerebrospinal fluid (CSF) flows based on the glymphatic system in the brain parenchyma without the lymphatic system. Increasing the collision frequency and attachment level between ADCs and cancer antigens in solid tumor parenchyma can be accomplished by (a) increasing the number of ADC molecules, (b) increasing the ADC molecular size, or (c) rendering solid tumor parenchyma leakier.

#### 2.7.1. Approaches That Increase the Number of ADC Molecules in Solid Tumor Parenchyma

(a) Blocking the salvation based on the FcRn by modifying the Fc region of ADCs might reduce the volume of distribution and eventually increase the population in solid tumor parenchyma, although their half-life would be shortened as a trade-off. Modifications to avoid the internalization at the endothelial cells via spontaneous endocytosis as a by-stander, leading lysosomal degradation into the degradation pathway, are also needed. Probabilistically, endocytosis of ADCs could be enhanced. Otherwise, raising the ADC dose increases the number of ADC molecules in solid tumor parenchyma based on the EPR effect.

#### 2.7.2. Approaches That Increase the ADC Molecular Size to Up the Probability of Collision

(b) mAb-loaded nanoparticles containing payloads are a type of PEG engager^EGFR^ methodology. The size of the designed compounds should be contained in endosomes to deliver payloads with the activity in the cytosol or the nucleus via RME. Endosomes induced from clathrin-dependent endocytosis are 85–150 nm in diameter. However, strictly speaking, this strategy does not use orthodox ADCs, due to the use of nanoparticles. The interaction of the nanodelivery methodology to RME machinery and the endosomal–lysosomal system and that of the ADC delivery methodology to RME machinery and the endosomal–lysosomal system are different from the point of view of structural pharmaceutical science, resulting in an alteration of the half-life, RME mechanisms, and payload release. Although mAb-loaded nanoparticles containing payloads effectively tether cancer antigens and are transported into cancer cells via RME, they raise the manufacturing cost. Nevertheless, mAb-loaded nanoparticles containing payloads are promising anti-cancer agents.

PEGylation, covalently attaching polyethylene glycol (PEG) chains to peptides, proteins, or nanoparticles, is expected to improve water solubility, non-immunization by decreasing macrophage clearance, non-filtration by increasing the molecular mass, and enzymatic non-degradation by interfering with enzymes [86]. Compounds with a diameter of less than 6–8 nm are subject to filtration and excretion by kidneys from the blood stream into urine [87]. Compounds of approximately 500 Da to approximately 1500 Da, such as glucuronic acid conjugates, are subject to secretion by the liver into bile over biliary epithelial cells. Thus, ADCs are not supposed to be filtered and excreted by the kidneys and liver. However, ADCs can be avoided through ingestion by the reticuloendothelial system, which comprises a network of cells and tissues such as the blood, spleen, bone marrow, liver, and lymph nodes. Macrophages, Kupffer cells in the liver, and microglial cells in the brain play such an ingestive role. Phagocytosis, a type of endocytosis, by phagocytes such as macrophages forms phagosomes (1–3 μm in diameter) [88,89]. Macrophages exhibit the highest attachment to particles with a longest dimension of 2–3 μm, which is the same as the size of common bacteria [90]. Owing to such phagosomes, bystander ADCs might happen to be phagocytosed together with substances unrelated to them, even though ADCs are not activated by binding to their antigens. For accidentally phagocytosed ADCs, so as not to become antigens, human or humanized mAbs should be used in ADCs. Nonetheless, the internalization of ADCs binding FcγRs into phagocytes or antigen-presenting cells via RME might be avoided by appropriate PEGylation. When too many PEGs are introduced into ADCs, such PEG chains might inhibit the binding of ADCs to cancer antigens on cancer cells. The Flory radiuses of PEG_100_, PEG_200_, PEG_300_, and PEG_400_ are approximately 5 nm, 8 nm, 11 nm, and 13 nm, respectively, where PEG_n_ would be n ethylene glycol repeats [91]. The radius is half of the diameter. PEG_200_ is almost as large as an IgG protein. Compared to nanoparticles, sizing up ADCs by PEGylation might be restricted due to molecular size, although a longer half-life and less immunogenicity were established [92]. Random PEGylation led to a loss of the biological potency of ADCs [93].

**Figure 7:**
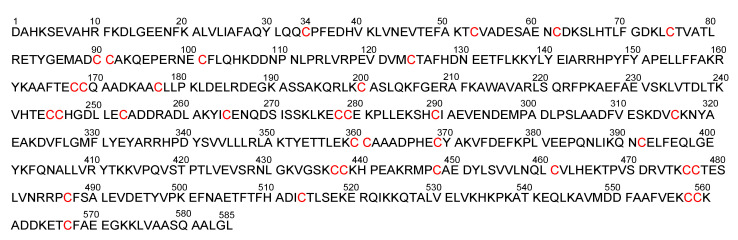
The sequence of human serum albumin 585 amino acids. There are 35 cysteine residues.

**Figure 8:**
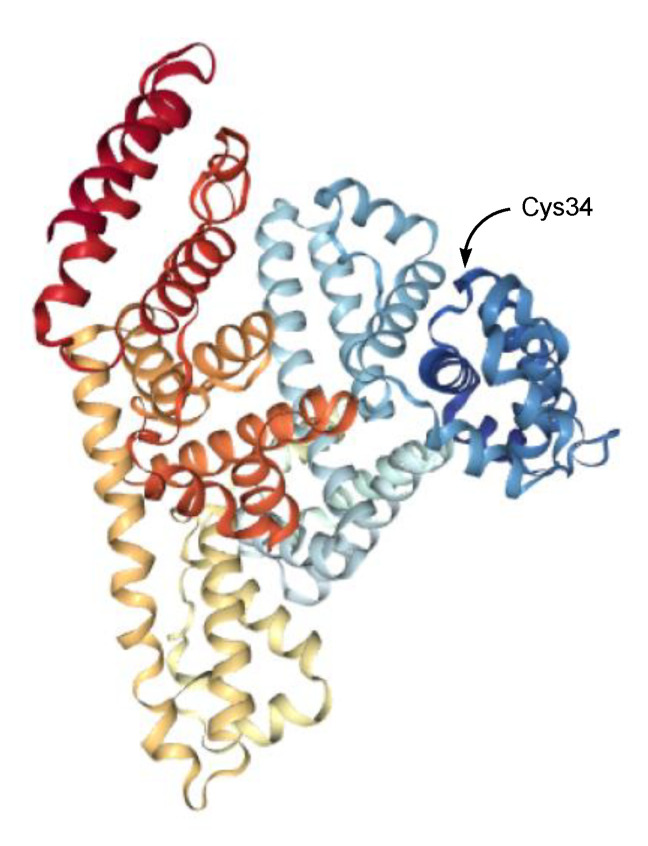
The X-ray crystal structure of human serum albumin (1BM0).

Albumin (approximately 7 nm in diameter [94], 65–70 kDa) is the most abundant circulating protein in plasma, accounts for 55–60% of all plasma proteins [95], and is used as a carrier for drug delivery [96]. Thus, ADCs modified to enhance their ability to bind albumin in serum can increase the ADC molecular size by forming complexes. In fact, an IgG–albumin complex through disulfide linking at the hinge region between the Fab and the Fc fragments of IgG in a 1:1 ratio was formed in normal human serum [97]. Such naturally volume-enlarged IgG–albumin complexes (approximately 21.2 nm in diameter, based on summation) gathered in solid tumor parenchyma based on the EPR effect might enhance endocytosis. Human serum albumin (Figure 7 and Figure 8) has only one free cysteine residue (Cys34) and 34 cysteine residues that form 17 intramolecular disulfide bonds [98]. Moreover, 70–80% of all serum albumins have the free sulfhydryl group of Cys34 as a reductive from [99]. Intravenously administered ADCs with PEG, at the tip of which the free sulfhydryl group is introduced, might more easily form heterodimers such as a PEGylated mAb–albumin complex (more than 21.2 nm in diameter) at albumin Cys34 due to the predominant population of Cys34-free albumins in serum among all serum proteins; in addition, homodimers such as PEGylated mAb–PEGylated mAb probably formed in drug preparation. Oxidative stress is involved in the pathophysiology of all cancers. It is considered that serum is exposed to oxidative stress through the endothelial fenestrations at neovasculatures [100], which would enhance disulfide bond formation. Although 2-iodoacetamide moiety can react irreversibly with the sulfhydryl group, it is uncertain which sulfhydryl groups react in the living body. Thus, reversible disulfide linking is safe in pharmacological treatment. The disulfide linking between the mAb–albumin complex would be cleaved by the disulfide bond reduction in endosomes, just as PE38 was cleaved from Moxetumomab pasudotox-tdfk in endosomes [26]. The anti-HER2 nanobody 11A4 fused to an albumin-binding domain (ABD) at their *C*-terminus demonstrated the internalization into cells in in vitro assay, irrespective of albumin presence, using HER2-expressing cells. 11A4-ABD-maleimide-auristatin F showed a greater anti-tumor efficacy after a single-dose administration in in vivo assay using HER2-positive NCI-N87 xenograft-bearing mice, compared to 11A4-maleimide-auristatin F without ABD [43]. It was suggested that albumin was relevant to the EPR effect [43].

#### 2.7.3. Approaches That Render Solid Tumor Parenchyma Leakier

**Figure 9:**
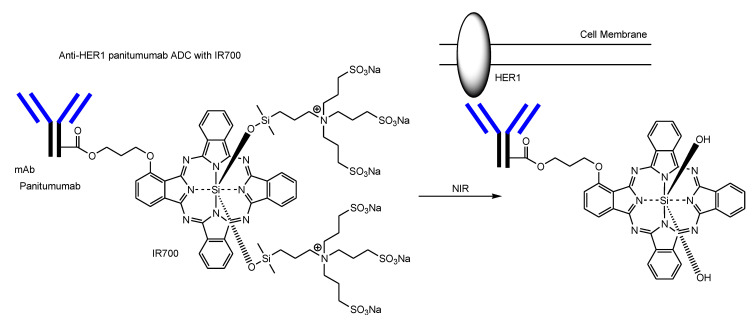
Anti-HER1 panitumumab ADC possessing IR700 with a drug-to-antibody ratio (DAR) of approximately 3 binds HER1 on the surface of cancer cells. At this time, the ADC–HER1 complex is activated by near-infrared (NIR) light (approximately 690 nm) and, consequently, releases its hydrophilic silanol units from IR700. Resulting hydrophobic complexes are aggregated and induce cytotoxicity by plasma membrane permeabilization.

(c) Solid tumors consist of the parenchyma and stroma. The systemic circulation and solid tumor stroma are virtually connected through a leaky vasculature. However, it is difficult for drugs to enter deeply into the inside of solid tumor parenchyma, because cancer cells are densely arranged there and the high pressure there prohibits drugs from advancing into the tumor. Moreover, it was thought that ADCs gathered in solid tumor parenchyma through a leaky vasculature based on the EPR effect were compelled to continue to remain due to little flow under static conditions (approximately 0 Pa), compared to the blood stream. If solid tumor parenchyma is leakier, ADCs could go through newly formed gaps along flows and could be endocytosed under low FSS. ADCs with IR700 can resolve this problem. IR700 is a dye that is activated by near-infrared (NIR) light (approximately 690 nm) and, consequently, injured the cell membrane. NIR light from the outside, peaking at 689 nm, can penetrate the living body to several centimeter depths from the surface without harmful side effects such as alteration of the immune system, unlike ultraviolet light. Thus, photodynamic therapy using IR700 will be a promising method for the treatment of solid cancers. Anti-HER1 panitumumab ADC possessing IR700 with a DAR of approximately 3 did not demonstrate tumor shrinkage after NIR light irradiation in in vivo assay using a tumor-xenografted mouse model bearing a 3T3/HER2 (HER1-negative) tumor in its dorsum, but it demonstrated tumor shrinkage after NIR light irradiation in in vivo assay using a tumor-xenografted mouse model bearing an A431 (HER1-positive) tumor in its dorsum [101]. When ADC–cancer antigen complexes are irradiated, they release their hydrophilic silanol units from IR700. As a result, such hydrophobic complexes are aggregated and make holes at the plasma membrane of cancer cells (Figure 9) [102]. With ADCs with IR700-induced cell rupture based on the membrane disruption, solid tumor parenchyma would be leakier than before. As a next strategy, the internal cytotoxicity by ADCs via RME could be effective. In the case of apoptosis, BAX/BAK oligomerization induces apoptotic pore formation at the mitochondrial outer membrane known as mitochondrial outer membrane permeabilization (MOMP), through which cytochrome c release into the cytoplasm is carried out [103]. Similarly, complexes of HER1 and ADC possessing IR700 without their hydrophilic silanol units might induce cytotoxic pore formation by their aggregation due to hydrophobic bonds or π–π stacking interactions. Eventually, alternative ADCs with payloads that show their activity in the cytoplasm or the nucleus could enter leakier solid tumor parenchyma.

### 2.8. Nanobody–Drug Conjugates

Although the Fc domain plays an important role in pharmacokinetic interactions with FcRs, IgG molecules are so large that they have problems such as membrane impermeability and their biotechnological production. Thus, nanobodies (4 nm and 2.5 nm in width and height, approximately 15 kDa) also known as VHH domains retain a specific binding ability to the corresponding antigens as Ab fragments and are expected to be a novel modality in pharmacotherapy [62]. Anti-transferrin receptor nanobodies with neurotensin, a type of ADC, was transported into the brain across the BBB via RMT [44]. Moreover, anti-EGFR nanobody–drug conjugates possessing two tandemly fused nanobodies (7D12 and 9G8) with MMAE exhibited potent anti-cancer activity in vivo in a solid tumor mouse model [104]. It is true that nanobody–drug conjugates are endocytosed but they do not benefit from the EPR effect in the systemic circulation due to their small size. Thus, nanobody–drug conjugates for cancer therapy should be devised to take the EPR effect. 11A4-ABD-maleimide-auristatin F that can bind to albumin is an interesting example [43]. Nanobody-loaded nanoparticles with payloads will be developed in this field. Anti-receptor nanobodies can act as vectors to trigger RME. Nanobodies targeting arbitrary proteins essential for cancer cells can act as payloads. Bispecific nanobodies targeting receptors and such proteins are pharmacokinetically and pharmacodynamically potent.

## 3. Conclusions

Chemotherapy has serious problems that carry a risk of side effects, such as membrane impermeability and wrong distribution. mAbs using IgG (approximately 14.2 nm in diameter, approximately 150 kDa) as a magic bullet exhibit highly selective antigen–antibody binding against cancer antigens. Nanocarriers (10–100 nm in diameter) are gathered in solid cancers based on the EPR effect (Figure 5). Payloads linked to ADCs can be delivered into cancer cells via RME and exhibit their activity after endosomal or lysosomal escape (Figure 2). Thus, ADCs with payloads through suitable linkers are promising anti-solid cancer pharmaceutical agents. Actually, 12 ADCs have been clinically approved by the FDA (Figure 3) (Table 1) [60]. This strategy using ADCs based on RME plays a vital role in cancer therapy and has been properly validated. Nonetheless, it has been a while since the last ADC was approved, although novel technologies such as nanobodies (15–45 kDa) known as single-domain Abs are being developed in basic research and clinical trials. However, ADCs using nanobodies might not be gathered in solid cancers based on the EPR effect due to molecular size. Thus, it is necessary to use a little ingenuity to improve canonical ADCs, although nanobody-loaded nanoparticles are thought to be gathered in solid cancers based on the EPR effect. (a) Increasing the number of ADC molecules, (b) increasing the ADC molecular size, or (c) rendering solid tumor parenchyma leakier can be done with current technology based on sustaining the innovation proselytized by Dr. Clayton Christensen.

(a) In order to increase the number of ADC molecules, increasing doses to deliver more ADCs into solid tumor parenchyma, or the modification of the Fc region to decrease the distribution volume by avoiding FcRn-mediated salvation, are relatively executable. (b) PEGs are used to enlarge the protein size and to improve water solubility. In fact, sacituzumab govitecan-hziy (Trodelvy^®^) has PEG_8_ in the linker [30,31,32]. However, the degree of PEGylation is thought to be restricted due to the size of an IgG molecule, because excess PEGs might counteract the nature of mAbs. On the other hand, it is known that an IgG–albumin complexes, through disulfide linking in a 1:1 ratio, are naturally formed in human serum [97]. Albumin is the largest protein component in serum and has a free sulfhydryl group at Cys34. Oxidative stress derived from solid cancers could enhance such disulfide bond formation. After accumulation based on the EPR effect, ADC–albumin complexes (approximately 21.2 nm in diameter, based on summation) would bind cancer antigens probabilistically to a greater degree than ADCs alone due to molecular size. Less hindered ADCs with PEGs whose terminals were each a free sulfhydryl group easily form a complex with albumin. Albumin is used as a carrier for drug delivery. Thus, the formation of ADC–albumin complexes is a potent method to enhance RME. (c) IR700 is a dye that is activated by NIR light (approximately 690 nm). The anti-HER1 panitumumab ADC with IR700 showed cytotoxic activity by NIR light irradiation after binding HER1 and, consequently, opened cracks in solid tumor parenchyma [101]. Such leakiness enables ADCs with payloads enter the deep region of cancer tissues. Effective cancer therapy by combination several approaches for ADCs to be endocytosed with a higher rate via RME could be accomplished.

Inspired readers are expected to suggest better ideas for solid cancer treatment than the present strategies and my proposals. In the future, in this research area, nanobody-loaded nanoparticles containing low-molecular payloads or ADCs with various formats of Ab fragments and their derivatives, such as the scFv, VHH domain, or nanobodies as payloads against target substances in solid cancer cells instead of low-molecular payloads, would be made with novel technology based on the disruptive innovation proselytized by Dr. Clayton Christensen.

Life phenomena are regulated by physically and biologically systematic structures based on the structuralism proselytized by Dr. Claude Lévi-Strauss. However, medicinal chemists and pharmaceutical scientists can act liberally and freely within machinery systems based on physically and biologically systematic structures in the living body, according to structural pharmaceutical science. Dr. Jean-Paul Sartre, according to existentialism, said: “Man is condemned to be free; because once thrown into the world, he is responsible for everything he does.” Thus, even though physically and biologically systematic structures are restricted and regulated, medicinal chemists and pharmaceutical scientists, for the meaning of their existence, can produce well-designed drugs that negotiate pharmacokinetically physical and biological machinery systems and, eventually, produce such systems pharmacodynamically by eliciting the activity with types of payloads. As a result, innovative pharmaceutical agents will be produced.

## Figures and Tables

**Table 1:** All compounds introduced in this perspective review.

#	Administrated Drug	Formulation	Cancer Antigen	Disease	Vector	Payload	Linker	Status	References
(1)	Anti-HER2 bispecific mAb	Anti-HER2 bispecific mAb targeting two non-overlapping epitopes on HER2	HER2	-	Anti-HER2 bispecific mAb	-	-	Basic research	[15]
(2)	Sym004	The mixture of two anti-EGFR mAbs	EGFR	-	anti-EGFR mAb	-	-	Basic research	[16]
(3)	Nivolumab (Opdivo^®^)	Anti-PD-1 mAb	PD-1	Metastatic lung squamous cell carcinoma	-	Nivolumab	-	Launched in 2014	[17]
(4)	Isatuximab (Sarclisa^®^)	Anti-CD38 mAb	CD38	Multiple myeloma	-	Isatuximab	-	Launched in 2020	[18]
(5)	Tafasitamab (Monjuvi^®^)	Anti-CD19 mAb	CD19	Diffuse large B cell lymphoma	-	Tafasitamab	-	Launched in 2020	[18]
(6)	Naxitamab (Danyelza^®^)	Anti-GD2 mAb	GD2	High-risk neuroblastoma and refractory osteomedullary disease	-	Naxitamab	-	Launched in 2020	[18]
(7)	Dostarlimab (Jemperli^®^)	Anti-PD-1 mAb	PD-1	Endometrial cancer	-	Dostarlimab	-	Launched in 2021	[18]
(8)	Gemtuzumab ozogamicin (Mylotarg^®^)	Anti-CD33 ADC with N-acetyl-γ-calicheamicin	CD33	Blood cancer	Gemtuzumab	N-acetyl-γ-calicheamicin	Acid-cleavable linker	Launched in 2000 and 2017	[19]
(9)	Brentuximab vedotin (Adcetris^®^)	Anti-CD30 ADC with MMAE	CD30	Blood cancer	Brentuximab	MMAE	Enzymatically cleavable linker	Launched in 2011	[20,21,22]
(10)	Adotrastuzumab emtansine (Kadcyla^®^)	Anti-HER2 ADC with DM1	HER2	Breast cancer	Adotrastuzumab	DM1	Uncleavable linker	Launched in 2013	[23,24]
(11)	Inotuzumab ozogamicin (Besponsa^®^)	Anti-CD22 ADC with N-acetyl-γ-calicheamicin	CD22	Blood cancer	Inotuzumab	N-acetyl-γ-calicheamicin	Acid-cleavable linker	Launched in 2017	[25]
(12)	Moxetumomab pasudotox-tdfk (Lumoxiti^®^)	Anti-CD22 ADC with PE38	CD22	Blood cancer	Moxetumomab	PE38	Reductively cleavable linker	Launched in 2018	[26]
(13)	Polatuzumab vedotin-piiq (Polivy^®^)	Anti-CD79b ADC with MMAE	CD79b	Blood cancer	Polatuzumab	MMAE	Enzymatically cleavable linker	Launched in 2019	[27]
(14)	Enfortumab vedotin-ejfv (Padcev^®^)	Anti-Nectin4 ADC with MMAE	Nectin4	Urothelial cancer	Enfortumab	MMAE	Enzymatically cleavable linker	Launched in 2019	[28]
(15)	Trastuzumab deruxtecan-nxki (Enhertu^®^)	Anti-HER2 ADC with DXd	HER2	Breast cancer	Trastuzumab	DXd	Enzymatically cleavable linker	Launched in 2019	[29]
(16)	Sacituzumab govitecan-hziy (Trodelvy^®^)	Anti-TROP2 ADC with SN-38	TROP2	Breast cancer	Sacituzumab	SN-38	Acid-cleavable linker	Launched in 2020	[30,31,32]
(17)	Belantamab mafodotin-blmf (Blenrep^®^)	Anti-BCMA ADC with MMAF	BCMA	Blood cancer	Belantamab	MMAF	Uncleavable linker	Launched in 2020	[33]
(18)	Loncastuximab tesirine-lpyl (Zynlonta^®^)	Anti-CD19 ADC with SG3199	CD19	Blood cancer	Loncastuximab	SG3199	Enzymatically cleavable linker	Launched in 2021	[34]
(19)	Tisotumab vedotin-tftv (Tivdak^®^)	Anti-Tissue Factor ADC withMMAE	Tissue Factor	Cervical cancer	Tisotumab	MMAE	Enzymatically cleavable linker	Launched in 2021	[35]
(20)	Datopotamab deruxtecan (Dato-DXd)	Anti-ROP2 ADC	ROP2	Solid cancer	Anti-ROP2 mAb	DXd	Linker	Clinical trial	[36]
(21)	Patritumab deruxtecan (HER3-DXd)	Anti-HER3 ADC	HER3	Solid cancer	Anti-HER3 mAb	DXd	Linker	Clinical trial	[37]
(22)	DS-7300	Anti-B7-H3 ADC	B7-H3	Solid cancer	Anti-HER3 mAb	DXd	Linker	Clinical trial	[38]
(23)	DS-6000	Anti-CDH6 ADC	CDH6	Solid cancer	Anti-CDH6	DXd	Linker	Clinical trial	[39]
(24)	DS-3939	Anti-TA-MUC1 ADC	TA-MUC1	Solid cancer	Anti-TA-MUC1	DXd	Linker	Clinical trial	[39]
(25)	BYON3521	Anti-c-MET receptor ADC	c-MET receptor	Solid cancer	Anti-c-MET receptor mAb	Duocarmycin	Cathepsin-cleavable linker	Phase1 (NCT05323045)	-
(26)	STRO-002	Anti-folate receptor α ADC	Folate receptor α	Solid cancer	Anti-folate receptor α mAb	3-Aminophenyl hemiasterlin	Cathepsin-cleavable linker	Phase1 (NCT03748186)	-
(27)	STI-6129	Anti-CD38 ADC	CD38	Solid cancer	Anti- CD38 mAb	Duostatin 5.2	Non-polyethylene glycol linker	Phase1 (NCT05584709)	-
(28)	ARX788	Anti-HER2 ADC	HER2	Solid cancer	Anti-HER2 mAb	MMAF	Non-natural amino acid linker	Phase2 (NCT04983121)	-
(29)	MORAb-202	Anti-folate receptor α ADC	Folate receptor α	Solid cancer	Anti-folate receptor α mAb	Eribulin	Cathepsin-cleavable linker	Phase2 (NCT05577715)	-
(30)	SYD985	Anti-HER2 ADC	HER2	Solid cancer	Anti-HER2 mAb	Duocarmycin	Cathepsin-cleavable linker	Phase2 (NCT04205630)	-
(31)	RC48 (disitamab vedotin)	Anti-HER2 ADC	HER2	Solid cancer	Anti-HER2 mAb	Auristatin E	Cathepsin-cleavable linker	Phase2 (NCT04329429)	-
(32)	MRG002	Anti-HER2 ADC	HER2	Solid cancer	Anti-HER2 mAb	MMAE	Cathepsin-cleavable linker	Phase2 (NCT05263869)	-
(33)	XMT-1536 (upifitamab rilsodotin)	Anti-NaPi2b ADC	NaPi2b	Solid cancer	Anti-NaPi2b mAb	Auristatin F	Hydrophilic polymer linker	Phase3 (NCT05329545)	-
(34)	IMGN-853 (mirvetuximab soravtansine)	Anti-folate receptor α ADC	Folate receptor α	Solid cancer	Anti-folate receptor α mAb	DM4	Disulfide-containing cleavable linker	Phase3 (NCT04296890)	-
(35)	Doxil^®^	Doxorubicin-encapsulated liposome coated with PEG	-	Ovarian cancer and breast cancer	-	Doxorubicin	-	Launched in 1999 and 2003	[40,41]
(36)	PEG engagerEGFR, Doxisome	Anti-EGFR and anti-PEG bispecific Ab, PEGylated liposomes containing doxorubicin	EGFR	Solid cancer	Anti-EGFR and anti-PEG bispecific Ab	Doxorubicin	-	Basic research	[42]
(37)	Anti-HER2 nanobody 11A4 fused to an albumin-binding domain-maleimide-auristatin F	Anti-HER2 nanobody 11A4 fused to an albumin-binding domain with auristatin F	HER2	Solid cancer	Anti-HER2 nanobody 11A4	Auristatin F	Maleimide	Basic research	[43]
(38)	Anti-transferrin receptor nanobodies with neurotensin	Anti-transferrin receptor nanobodies with neurotensin	-	-	Anti-transferrin receptor nanobodies	Neurotensin	-	Basic research	[44]
(39)	Anti-EGFR nanobodies-drug	Anti-EGFR nanobodies with MMAE	EGFR	Solid cancer	Anti-EGFR nanobodies	MMAE	-	Basic research	-
(40)	ADC–albumin complex	ADC with or without PEGs	Arbitrary	Solid cancer	Arbitrary	Arbitrary	-	Under analysis in Tashima lab	-
(41)	mAb-loaded nanoparticles containing payloads	mAb-loaded nanoparticles containing payloads	Arbitrary	Solid cancer	Arbitrary	Arbitrary	-	Under analysis in Tashima lab	-
